# COVID-19 coagulopathy: is it disseminated intravascular coagulation?

**DOI:** 10.1007/s11739-020-02601-y

**Published:** 2020-12-24

**Authors:** Marcel Levi, Toshiaki Iba

**Affiliations:** 1 Department of Medicine, University College London Hospitals NHS Foundation Trust, 250 Euston Road, London, NW1 2PG UK; 2 Cardiometabolic Programme-NIHR UCLH/UCL BRC, London, UK; 3 Department of Emergency and Disaster Medicine, Jutendo University Graduate School of Medicine, Tokyo, Japan

**Keywords:** COVID-19, SARS-CoV-2, Coagulation, Thrombosis, Pulmonary embolism, d-dimer, Disseminated intravascular coagulation, Thrombotic microangiopathy

## Abstract

One of the significant complications of severe COVID-19 infections is a coagulopathy that seems to be related to the occurrence of venous and arterial thromboembolic disease. The coagulation changes mimic but are not identical to disseminated intravascular coagulation (DIC). The vast majority of patients with COVID-19 do not meet the criteria for usual forms of DIC. In addition, there seem to be features of a strong local pulmonary thrombotic microangiopathy and direct endothelial cell infection and injury by the virus that affect the coagulopathic response to severe COVID-19. It seems COVID-19 leads to a distinct intravascular coagulation syndrome that may need separate diagnostic criteria.

## Introduction

A COVID-19 infection begins when SARS-CoV-2 virus, is transmitted from one human to another, via oral ingestion of virus-containing droplets or inhalation. The virus binds to the epithelium in the nasal or oral cavity via the angiotensin converting enzyme-2 (ACE2) receptor. Initially there is local replication of the virus which evokes a limited innate immune response, but at this stage infected people can already infect others [1]. The virus propagates and tracks down the respiratory tract and the airways, and a more vigorous innate immune response is initiated, associated with the occurrence of systemic pro-inflammatory cytokines and activated immune cells. At this moment, COVID-19 disease becomes clinically apparent with usually self-limiting minor to moderate symptoms of a respiratory infection and constitutional symptoms such as fever, myalgia and fatigue. However, in about a fifth of the patients the virus will infect more distal alveolar cells, once again via the ACE2 receptor, causing pulmonary infiltrates and acute lung injury. This may culminate in potentially very severe disease requiring mechanical ventilation and is associated with significant mortality [2].

Patients with severe forms of COVID-19 display coagulation abnormalities that have been associated with respiratory deterioration and death [3, 4]. In addition, many patients with severe COVID-19 infections develop venous thromboembolism, which appears to be related to the coagulopathy [5, 6]. It has repeatedly been confirmed that pulmonary embolism contribute to a sudden deterioration of pulmonary oxygen exchange that is occasionally seen in patients with COVID-19 infections.

The coagulopathy associated with COVID-19 mimics other systemic coagulopathies that are regularly seen in severe infections, most notably disseminated intravascular coagulation (DIC) [7]. However, COVID19 has specific clinical and laboratory features that are distinctly different from the ‘classical’ presentation of DIC [8, 9].

## COVID-19 coagulopathy

The most remarkable abnormal coagulation assay in severe COVID-19 patients is an excessively elevated D-dimer level [3, 4, 10]. A large initial COVID-19 series found abnormally elevated D-dimer levels in 46% of all cases (43% in non-severe patients versus 60% in critically ill ICU patients [11]. In another study increased levels of D-dimer were related to a poor outcome [3]. In an investigation of 343 patients it was demonstrated that D-dimer levels of over 2.0 mg/L predicted mortality with a sensitivity of 92% and a specificity of 83% [12].

In patients with more severe COVID-19 infection the prothrombin time (PT) is only moderately increased (about 3 s) [4]. Prolongation of the activated partial thromboplastin time (aPTT) is less frequently seen although this could be hidden by high levels of factor VIII and fibrinogen that in some cases affect the aPTT.

Another hemostatic defect that is seen in the most severely affected patients is thrombocytopenia. [13, 14] The majority of patients have a platelet count between 100 and 150 × 10^9^/L and more severe thrombocytopenia is hardly (< 5%) seen [10, 11]. A meta-analysis demonstrated significantly lower platelet counts (about minus 30 × 10^9^/L (95% confidence interval [CI] − 35 to − 29 × 10^9^/L) in critically ill COVID-19 and thrombocytopenia defined as below the lower limit of the reference range was associated with more than fivefold higher risk of severe disease (odds ratio 51.1, 95% CI 1.8–14.6) [15]. In contrast to low platelet counts seen in other severe infections, thrombocytopenia in COVID-19 has not been significantly associated with mortality [13].

Coagulation factor levels in COVID19 are usually within the normal range but mean fibrinogen plasma concentration (and to a lesser extent factor VIII:c) can be remarkably high, which is presumably due to an acute phase response [4]. In very ill patients a rapid decline in plasma fibrinogen levels below < 1.0 g/L was seen shortly before they died. Plasma levels of physiological anticoagulants such as protein C and antithrombin are mildly low, in particular in the non-surviving patients, but these levels rarely decrease below 80% of normal concentrations [4]. Several studies have reported abnormal overall clotting and increased viscoelastic parameters by thromboelastography [16].

## Pathogenesis and relevance of COVID-19 coagulopathy

In severe COVID-19 systemic levels of pro-inflammatory cytokines, such as tumor necrosis factor-α (TNF-α) and interleukin (IL)-1 and IL-6, are markedly increased [10]. IL-6 induces tissue factor expression on monocytes and macrophages, which consequently leads to thrombin generation. In a subset of most severely affected COVID-19 patients, a cytokine ‘storm’ can be detected, characterized by high levels of pro-inflammatory cytokines and chemokines [9].

Coronavirus infections are associated with a remarkable fibrinolytic profile. Experiments in mice with a targeted deletion of the urokinase-type plasminogen gene pointed to a urokinase-driven route as an important factor in mortality [17]. In addition, in patients with human SARS-CoV-1 infection plasma concentrations of tissue-type plasminogen activator (t-PA) were sixfold higher than normal [18]. It is probable that inflammation-driven endothelial cell perturbation results in substantial release of plasminogen activators which clarifies the high levels of d-dimer in the most severely affected COVID-19 patients. Also, plasmin effects on metalloproteinases can result in extracellular matrix modification expediting capillary leakage and lung edema. Of note, the effects on plasminogen activators do not translate into a hyperfibrinolytic state or an increased risk of systemic bleeding in patients with COVID-19.

There is a marked relationship between bronchoalveolar coagulation and fibrinolysis and the development of acute respiratory distress syndrome (ARDS), in which intrapulmonary fibrin deposition as a result of deranged bronchoalveolar fibrin turnover is a critical step. The clinical and laboratory picture of serious ARDS in ventilated COVID-19 patients and important coagulation abnormalities may point to a potential role of bronchoalveolar fibrin turnover in the most severe COVID-19 patients.

The coagulation changes associated with COVID-19 infection point in the direction of a hypercoagulable state that may at least cause an enhanced risk of thromboembolic complications. Immobilization and vascular damage are other factors that may increase the risk of thrombosis. A large number of clinical observational studies in almost 2000 patients point to an incidence of venous thromboembolism of up to 35% in patients with severe COVID-19. Several retrospective studies point to a higher risk of venous thromboembolism in patients with more severe COVID-19 coagulopathy. It has been suggested that (low molecular weight) heparin prophylaxis can reduce the risk of venous thromboembolism. In addition, there is ample experimental and some clinical evidence that heparin has antithrombotic and anti-inflammatory properties that may be relevant for treatment of ‘inmmunothrombosis’ [19].

The relevance of microvascular thrombosis for organ dysfunction has also been suggested based on post-mortem pathological reports. Several reports highlight vascular wall thickening, stenosis of the vascular lumen, and microthrombus formation associated with findings of ARDS. Comparable pathological observations are made in the vasculature of other organs [20].

Taken together, it seems there are two parallel clinical manifestations of the COVID-19 coagulopathy: (1) ‘classic’ venous thromboembolism (presumably provoked by cytokine-mediated activation of coagulation in combination with other risk factors for thrombosis) and (2) diffuse microthrombosis with endothelial damage (in the lungs) directly caused by the coronavirus.

## Is this DIC?

In 2001 the International Society on Thrombosis and Haemostasis proposed that DIC could be defined as “an acquired syndrome characterized by the intravascular activation of coagulation with loss of localization arising from different causes”. The COVID-19 coagulopathy seems to meet this definition. Also, the combination of increased D-dimer, low platelet counts, and (slightly) prolonged coagulation times (such as the PT) reminds of the abnormalities commonly seen in DIC [7]. However, there are marked differences between the COVID-19 coagulopathy and the DIC commonly seen in patients with severe infections and sepsis [21]. Usually in sepsis-associated DIC a more severe thrombocytopenia is observed. Also, these patients have commonly much lower levels of clotting factors and marked decreased plasma concentrations of coagulation inhibitors, such as antithrombin and protein C.

Interestingly, not many patients with severe COVID-19 fulfill the criteria for DIC of the International Society on Thrombosis and Haemostasis [9, 22]. Most reports indicate that according to these criteria the incidence of DIC is about 5% in COVID-19 non-survivors and 0% in survivors. Also, the clinical manifestation of the COVID-19 coagulopathy is mostly prothrombotic in the absence of a real consumption coagulopathy, with a high rate of venous (and possibly arterial) thromboembolism, and not many hemorrhagic complications.

Based on all this, the COVID-19 cannot be classified as a usual manifestation of DIC and should be categorized as a specific form of an intravascular coagulation syndrome that may need new diagnostic criteria. Also, (adjunctive) therapeutic interventions to be tested for this coagulopathy needs to take into account this distinction, moreover as there is currently no specific therapy for most common forms of DIC.

## Alternative explanations for the COVID-19 coagulopathy

**Fig. 1:**
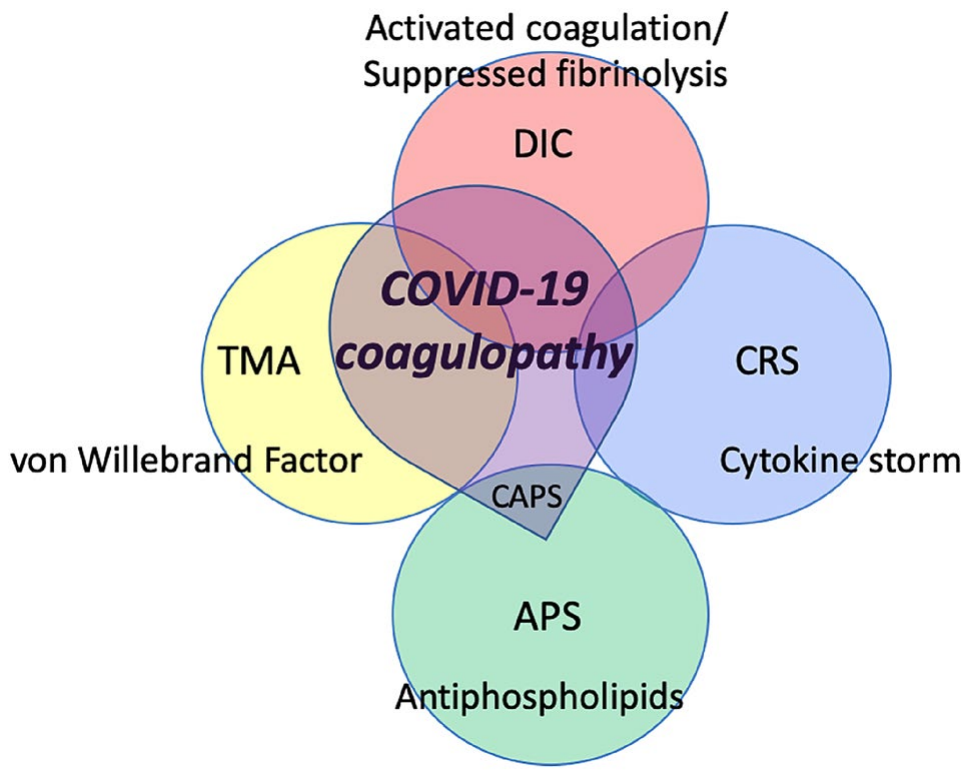
The characteristics of COVID-19-associated coagulopathy. COVID-19-associated coagulopathy partially overlaps with infection-induced disseminated intravascular coagulation (DIC), cytokine release syndrome (CRS), antiphospholipid syndrome (APS), and thrombotic microangiopathy (TMA), however, it does not meet the criteria for any of these coagulopathies

COVID-19 overlaps with various other coagulopathies (Fig. 1). Some features mimic cytokine release syndromes and in a number of studies positive antiphospholipid antibodies have been reported (but never confirmed according to current guidelines for antiphospholipid syndrome classification) [23]. Histopathology from post-mortem examinations in COVID-19 patients have shown typical microvascular platelet-rich thrombotic depositions in small vessels of the lungs along foci of local hemorrhage and accumulation and entrapment of inflammatory cells, such as neutrophils, in alveolar capillaries. This picture is compatible with pulmonary thrombotic microangiopathy [24]. Thrombotic microangiopathy is a result of increased platelet adhesion to the vascular endothelium in association with platelet aggregation and activation causing consumptive thrombocytopenia [25]. The resultant platelet thrombi in the microvasculature cause impaired organ function and classically contribute to complications such as renal insufficiency or neurological disease as well as microangiopathic hemolysis. The presence of (at least localized) thrombotic microangiopathy is supported by observations of abnormal von Willebrand factor/ADAMTS-13 ratios. A recent report also correlates low ADAMTS13 plasma levels with mortality in seriously ill COVID-19 patients [26]. However, there is at present insufficient evidence to support the presence of systemic thrombotic microangiopathy.

The striking increase in vascular complications and specific coagulation changes as discussed above seem to point to a specific endothelial cell involvement in COVID-19. COVID-19 (and other coronavirus infections) are clear examples of direct viral infection of endothelial cells [27]. Indeed, infection- and inflammation-induced endothelial cell perturbation and injury can provide an excellent scaffold for intravascular thrombus formation. It may also may cause increased platelet-vessel wall interaction, due to release of high molecular weight multimers of von Willebrand factor, insufficiently cleaved by deficient ADAMTS13, and resulting in thrombotic microangiopathy in the microvasculature [28].

## Conclusion

The most severe COVID-19 infections cause coagulation abnormalities that remind of DIC but is distinctly different from its usual presentation and does not meet international criteria for DIC. However, COVID-19 appears to result in a specific coagulopathy that is more localized and prothrombotic (rather than hemorrhagic), with elements of in situ thrombotic microangiopathy and direct endothelial infection and injury. The COVID-19 coagulopathy should be classified as a distinct manifestation of an intravascular coagulation syndrome that may need new diagnostic criteria.

## References

[1] Mason RJ (2020). Pathogenesis of COVID-19 from a cell biology perspective. Eur Respir J.

[2] Zhou P, Yang XL, Wang XG (2020). A pneumonia outbreak associated with a new coronavirus of probable bat origin. Nature.

[3] Zhou F, Yu T, Du R (2020). Clinical course and risk factors for mortality of adult inpatients with COVID-19 in Wuhan, China: a retrospective cohort study. Lancet.

[4] Tang N, Li D, Wang X, Sun Z (2020). Abnormal coagulation parameters are associated with poor prognosis in patients with novel coronavirus pneumonia. J Thromb Haemost.

[5] Klok FA, Kruip M, van der Meer NJM (2020). Incidence of thrombotic complications in critically ill ICU patients with COVID-19. Thromb Res.

[6] Marietta M, Coluccio V, Luppi M (2020). COVID-19, coagulopathy and venous thromboembolism: more questions than answers. Intern Emerg Med.

[7] Gando S, Levi M, Toh CH (2016). Disseminated intravascular coagulation. Nature Rev Dis Prim.

[8] Levi M (2020). COVID-19 coagulopathy vs disseminated intravascular coagulation. Blood Adv.

[9] Levi M, Thachil J (2020). Coronavirus disease 2019 coagulopathy: disseminated intravascular coagulation and thrombotic microangiopathy-either, neither, or both. Semin Thromb Hemost.

[10] Huang C, Wang Y, Li X (2020). Clinical features of patients infected with 2019 novel coronavirus in Wuhan. China Lancet.

[11] Guan WJ, Ni ZY, Hu Y (2020). Clinical characteristics of coronavirus disease 2019 in China. N Engl J Med.

[12] Zhang L, Yan X, Fan Q (2020). d-dimer levels on admission to predict in-hospital mortality in patients with Covid-19. J Thromb Haemost.

[13] Lippi G, Plebani M, Henry BM (2020). Thrombocytopenia is associated with severe coronavirus disease 2019 (COVID-19) infections: a meta-analysis. Clin Chim Acta.

[14] Brogaard Larsen J, Pasalic L, Hvas AM. Platelets in Coronavirus Disease. Seminars in Thrombosis & Hemostasis 2020. 10.1055/s-0040-1710006 PMC7645810 32356294

[15] Favaloro EJ, Lippi G (2020). Maintaining Hemostasis and Preventing Thrombosis in Coronavirus Disease 2019 (COVID-19)-Part I. Semin Thromb Hemost.

[16] Ranucci M, Ballotta A, Di Dedda U (2020). The procoagulant pattern of patients with COVID-19 acute respiratory distress syndrome. J Thromb Haemost.

[17] Gralinski LE, Bankhead A, Jeng S (2013). Mechanisms of severe acute respiratory syndrome coronavirus-induced acute lung injury. mBio.

[18] Liu ZH, Wei R, Wu YP (2005). Elevated plasma tissue-type plasminogen activator (t-PA) and soluble thrombomodulin in patients suffering from severe acute respiratory syndrome (SARS) as a possible index for prognosis and treatment strategy. Biomed Environ Sci BES.

[19] Buijsers B, Yanginlar C, Maciej-Hulme ML, de Mast Q, van der Vlag J (2020). Beneficial non-anticoagulant mechanisms underlying heparin treatment of COVID-19 patients. EBioMedicine.

[20] Iba T, Levy JH, Levi M, Thachil J (2020). Coagulopathy in COVID-19. J Thromb Haemost.

[21] Coccheri S (2020). COVID-19: The crucial role of blood coagulation and fibrinolysis. Intern Emerg Med.

[22] Deng Y, Liu W, Liu K (2020). Clinical characteristics of fatal and recovered cases of coronavirus disease 2019 in Wuhan, China: a retrospective study. Chin Med J (Engl).

[23] Miyakis S, Lockshin MD, Atsumi T (2006). International consensus statement on an update of the classification criteria for definite antiphospholipid syndrome (APS). J Thromb Haemost.

[24] Fox SE, Atmakbekov A, Harbert JL, Li G, Brown JQ, Vander Heide RS (2020). Pulmonary and cardiac pathology in Covid-19: the first autopsy series from New Orleans. MedRxiv.

[25] George JN, Nester CM (2014). Syndromes of thrombotic microangiopathy. N Engl J Med.

[26] Bazzan M, Montaruli B, Sciascia S, Cosseddu D, Norbiato C, Roccatello D (2020). Low ADAMTS 13 plasma levels are predictors of mortality in COVID-19 patients. Intern Emerg Med.

[27] Varga Z, Flammer AJ, Steiger P, et al. Endothelial cell infection and endotheliitis in COVID-19. Lancet 2020. 10.1016/S0140-6736(20)30937-5 PMC7172722 32325026

[28] Levi M, Scully M, Singer M (2018). The role of ADAMTS-13 in the coagulopathy of sepsis. J Thromb Haemost.

